# DUT (p.Y116C)-Mutation-Induced Thrombocytopenia in Rabbits

**DOI:** 10.3390/ijms26094169

**Published:** 2025-04-28

**Authors:** Mengmeng Fang, Shujun Yang, Ruonan Liu, Xinyu Wu, Liqiang Jiang, Jie Yang, Xin Liu, Gerong Wang, Chao Mu, Xiuwen Wang, Yuning Song

**Affiliations:** 1 State Key Laboratory for Diagnosis and Treatment of Severe Zoonotic Infectious Diseases, Key Laboratory for Zoonosis Research of the Ministry of Education, and College of Veterinary Medicine, Jilin University, Changchun 130062, China; fangmm22@mails.jlu.edu.cn (M.F.); yangsc23@jlu.edu.cn (S.Y.); liurn9917@jlu.edu.cn (R.L.); wuxinyu20@mails.jlu.edu.cn (X.W.); jianglq22@mails.jlu.edu.cn (L.J.); yangj0060@163.com (J.Y.); liux20@mails.jlu.edu.cn (X.L.); wanggr23@jlu.edu.cn (G.W.); muchao24@mails.jlu.edu.cn (C.M.); 2 College of Life Sciences, Jilin Normal University, No. 1301, Hai Feng Street, Tiexi District, Siping 136000, China; liaoshiwxw@163.com

**Keywords:** thrombocytopenia, DUT, rabbit model, mitochondria, mitophagy

## Abstract

Thrombocytopenia is a hematologic disorder characterized by an abnormally low platelet count in peripheral blood. Recent studies have identified mutations in DUT as the primary cause of bone marrow failure and diabetes mellitus syndrome (BMFDMS), a condition commonly associated with thrombocytopenia. In this study, a novel rabbit model of thrombocytopenia carrying the DUT c.3020A>G (p.Y116C) mutation was established using SpRY-ABEmax-mediated base editing. This model accurately recapitulates the clinical manifestations of human thrombocytopenia. Phenotypic analysis has revealed that mutant rabbits exhibited significant reductions in megakaryocyte numbers, platelet counts, and survival rates when compared to wild-type controls. Mechanistic investigations showed that the DUT mutation leads to mitochondrial structural abnormalities and functional impairments. Notably, platelets from DUT (p.Y116C)-mutant rabbits exhibited markedly reduced DUT protein expression and enhanced mitophagy, potentially mediated through the Park2 pathway. This study presents the first genetic model of thrombocytopenia that closely mimics the human DUT (p.Y116C) mutation, offering new insights into the relationship between DUT mutations and platelet function, and highlighting potential therapeutic targets for human thrombocytopenia.

## 1. Introduction

Thrombocytopenia is a hematologic disorder characterized by an abnormally low platelet count in the peripheral blood [1]. Hereditary thrombocytopenia typically manifests with an early onset, often accompanied by megakaryocytopenia or the complete absence of megakaryocytes, leading to progressive bone marrow failure. The molecular pathogenesis of this condition is often linked to specific genetic mutations.

Mitochondria are essential for maintaining platelet function and longevity [1,2]. Mitochondrial dysfunction with abnormal respiratory chain electron transfer and adenosine triphosphate (ATP) energy generation in platelets causes reduced ATP production, the generation of mitochondrial reactive oxygen species (ROS), and even programmed cell death. Mitochondrial homeostasis is tightly regulated to ensure optimal mitochondrial number and appropriate quality to sustain physiological functions [3,4,5]. For instance, mitochondria undergo continuous fission and fusion cycles, providing mechanisms for repairing or diluting defective mitochondria [2,6]. Severely damaged mitochondria beyond repair are segregated from the mitochondrial network by mitochondrial fission, and are eliminated through mitochondrial autophagy (removal of mitochondria) or apoptosis (removal of cells) [7]. Mitophagy, a selective process targeting damaged mitochondria for lysosomal degradation, is considered a crucial pathway in both yeast and mammalian cells [8,9,10,11], with Parkin/PINK1 (phosphatase and tensin homolog-induced putative kinase 1)-mediated mitophagy being one of the primary mechanisms [12,13,14,15].

In 2017, a novel syndrome characterized by bone marrow failure, diabetes mellitus, erythroid lineage abnormalities, and thrombocytopenia was first reported. It was the first report of a DUT mutation found in human patients. The study described four patients from two unrelated families diagnosed with this syndrome. Genetic analysis revealed that the mutation of DUT (chr15:g.48,626,619A>G, hg19), affecting both the mitochondrial (DUT-M p.Y142C) and the nuclear (DUT-N p.Y54C), was the underlying cause of this condition [16]. In 2022, four more patients with homogeneic isosite mutations were identified [17]. The DUT gene encodes deoxyuridine triphosphate nucleotide hydrolase (dUTPase), a critical enzyme in DNA synthesis. dUTPase catalyzes the hydrolysis of dUTP into dUMP and pyrophosphate, thereby preventing uracil misincorporation into DNA. Additionally, this enzyme regulates the intracellular dUTP/dTTP ratio. An elevated dUTP/dTTP ratio can lead to repeated dUTP incorporation, triggering a futile repair cycle that induces single- and double-stranded DNA breaks, ultimately resulting in cell death [18]. Despite the critical role of DUT as an essential gene, its specific association with thrombocytopenia remains poorly understood.

Animal models are essential for understanding of the pathogenesis and treatment of the disease [19]. Compared to mice, rabbits exhibit better genetic similarity to humans, circulate more blood, and have a longer lifespan than mice. Additionally, the application of advanced genome editing tools, such as the SpRY-ABEmax editor used in this study, enables the precise replication of human pathogenic mutations. This editor has demonstrated remarkable efficiency in A-to-G base editing, as evidenced by its high performance in our prior rabbit studies [20,21,22]. Therefore, rabbits serve as an ideal model for investigating DUT-mutation-induced thrombocytopenia.

In this study, we successfully generated a rabbit model carrying the DUT c.3020A>G (p.Y116C) mutation, which corresponds to the human DUT gene mutation (chr15: g.48,626,619A>G, hg19). This model not only replicates the disease phenotype, but also provides critical mechanistic insights into the pathogenesis of DUT (p.Y116C)-mediated thrombocytopenia. This model represents a valuable platform for the further investigation of the disease, and the development of novel diagnostic and therapeutic strategies.

## 2. Results

### 2.1. Generation of DUT (p.Y116C) Rabbits of Thrombocytopenia Using SpRY-ABEmax

**Figure 1:**
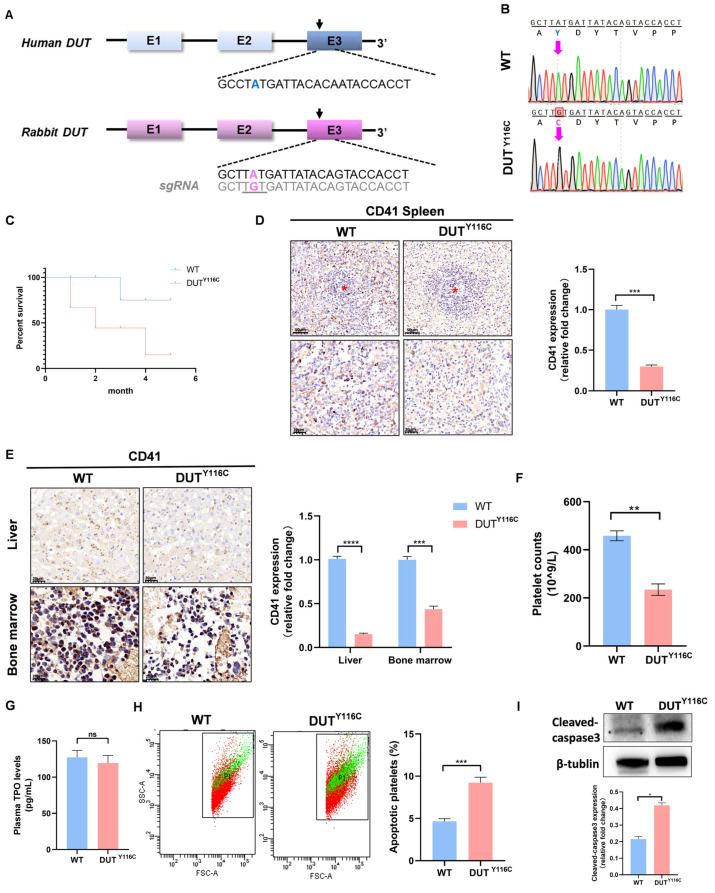
Generation of DUT (p.Y116C) rabbits of thrombocytopenia using SpRY-ABEmax. (**A**) Schematic of the sgRNA target site at the human and rabbit DUT gene locus. The grey color indicates the sgRNA sequence of the rabbit DUT gene. The position of the point mutation is indicated in pink. (**B**) Sanger sequencing results of DUT gene mutations of two genotypes in rabbit pups. The pink arrows point to mutant base sites. (**C**) Survival curves of age-matched WT controls (*n* =  7) and DUT (p.Y116C) rabbits (*n* = 7). (**D**) Immunohistochemical images of CD41 in the spleens of WT and DUT (p.Y116C) rabbits. The scale bars represent 20 μm and 50 μm (*n* ≥ 3; values are the means ± SEM, *** *p* < 0.001). The red asterisks indicate the splenic corpuscles in the histological sections. (**E**) Immunohistochemical images of CD41 in the liver and bone marrow of WT and DUT (p.Y116C) rabbits. Scale bars, 20 μm (*n* ≥ 3; values are the means ± SEM, *** *p* < 0.001, **** *p* < 0.0001). (**F**) Platelet counts in WT and DUT (p.Y116C) rabbits (*n* ≥ 3; values are the means ± SEM, ** *p* < 0.01). (**G**) The level of TPO in the plasma of WT rabbits and DUT rabbits (*n* ≥ 3; values are the means ± SEM, ns, not significant). (**H**) Platelet apoptosis was measured by annexin V staining using flow cytometry. The statistics for the platelet apoptosis in sections WT and DUT (p.Y116C) rabbits are shown (*n* ≥ 3; values are the means ± SEM, *** *p* < 0.001). (**I**) Cleaved-caspase3 levels were tested in the platelets of DUT (p.Y116C) rabbits and control rabbits by Western blot analysis. The grayscale values of all the bands were measured with ImageJ V1.8.0.112 software and the statistical data are illustrated (*n* ≥ 3; values are the means ± SEM, * *p* < 0.05).

The DUT (p.Y116C) rabbit model was constructed using SpRY-ABEmax. A single-guide RNA (sgRNA) targeting the c.3020A>G (p.Y116C) mutation in exon 3 of the DUT gene was designed (Figure 1A). To evaluate the efficacy of SpRY-ABEmax-mediated base editing in rabbit zygotes, 160 injected embryos were transferred to four pseudo-pregnant recipient rabbits. The gene targeting efficiency was assessed, revealing that 10/20 embryos (50%) carried the DUT (p.Y116C) mutation, with 4/20 embryos (20%) exhibiting homozygous mutations. Two recipient mothers successfully carried their pregnancies to term and gave birth to 10 live pups. The genotyping of the pups revealed that seven (70%) carried the DUT mutation, including six homozygous DUT (p.Y116C) mutations (Figure 1B) and one frameshift mutation (Figure S1A). Notably, the DUT (p.Y116C) rabbits exhibited significantly reduced survival rates within six months compared to wild-type (WT) controls (Figure 1C).

Platelets are derived from mature megakaryocytes (MKs), and the spleen is a primary site for MKs [23] in rabbits. Immunohistochemical analysis of spleen sections from WT and DUT (p.Y116C) rabbits revealed a significant reduction in the number of CD41+ MKs in the mutant rabbits compared to the age-matched WT controls (Figure 1D). Similarly, fewer MKs were observed in the bone marrow (BM) and liver of DUT (p.Y116C) rabbits relative to the WT controls (Figure 1E). Peripheral blood analysis showed that the platelet count in DUT (p.Y116C) rabbits was 234.5 ± 23.574 × 10^9^ circulating platelets/L, compared to 458.25 ± 20.597 × 10^9^ in WT rabbits (Figure 1F). These results demonstrated that a DUT (p.Y116C) mutation could cause thrombocytopenia in rabbits. No significant differences in red blood cell (RBC) and white blood cell (WBC) counts were observed between mutant and WT rabbits (Figure S1B). Furthermore, no significant differences in plasma thrombopoietin (TPO) levels were observed between mutant and WT rabbits (Figure 1G), suggesting that DUT primarily regulates megakaryocyte differentiation rather than TPO production. To assess platelet apoptosis, peripheral-blood-derived platelets were analyzed via annexin V staining and cleaved caspase-3 immunoblotting. Flow cytometry revealed a significant increase in annexin V-positive platelets in DUT (p.Y116C)-mutant rabbits compared to the controls (Figure 1H). Western blot (WB) further demonstrated elevated cleaved caspase-3 levels in mutant platelets relative to the wild type (WT) (Figure 1I). Collectively, these results demonstrate the successful generation of the thrombocytopenia rabbit model using the SpRY-ABEmax system.

### 2.2. DUT (p.Y116C) Is a Reduced Mutation and Is Highly Expressed in Megakaryocytes

**Figure 2:**
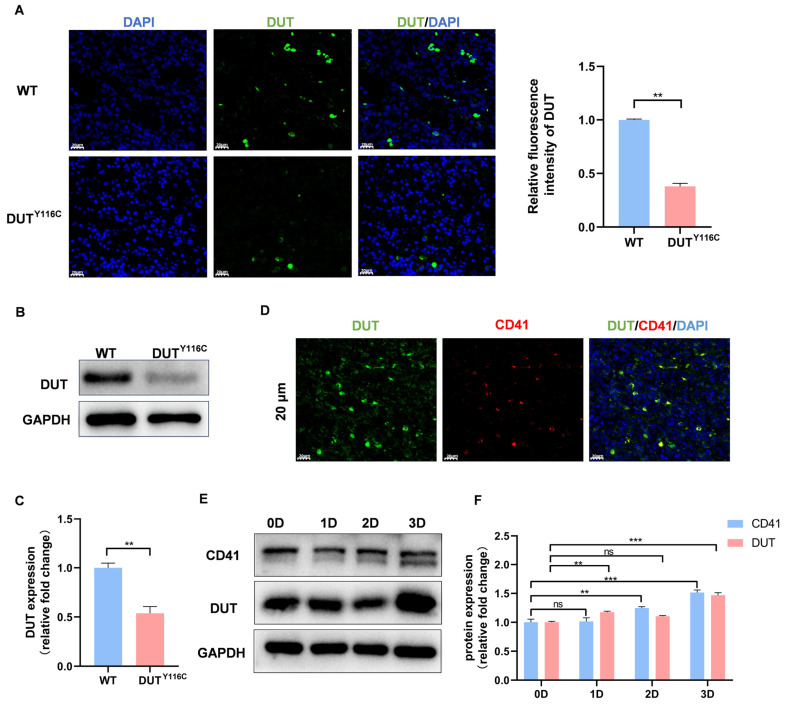
(**A**) DUT levels were tested in the spleen of DUT (p.Y116C) rabbits and control rabbits by immunofluorescence. The scale bars represent 20 μm (** *p* < 0.01). (**B**) DUT levels were tested in the platelets of DUT (p.Y116C) rabbits and control rabbits by Western blot analysis. (**C**) The grayscale values of all the bands were measured with ImageJ software and the statistical data are illustrated (*n* ≥ 3; values are the means ± SEM, ** *p* < 0.01). (**D**) Immunolabeling of DUT (green) and CD41 (red) in control rabbits. Megakaryocytes were stained with CD41 antibodies. Scale bars, 20 μm. (**E**,**F**) MEG-01 cells were treated with 100 nM PMA for 3 days to induce megakaryocyte differentiation. DUT and CD41 levels were tested in MEG-01 cells by Western blot analysis (*n* ≥ 3; values are the means ± SEM, ns, not significant; ** *p* < 0.01; *** *p* < 0.001).

Previous studies have demonstrated that cysteine mutations at position 142 (corresponding to position 116 in the rabbit DUT gene) significantly affect the stability of the nuclear isoform of human dUTPase. To investigate the impact of the mutation at amino acid position 116 on the DUT gene, immunofluorescence analysis revealed a significant reduction in DUT gene expression in the spleen of mutant rabbits (Figure 2A). Similarly, DUT expression was notably decreased in platelets from DUT (p.Y116C) rabbits (Figure 2B,C). Considering the link between thrombocytopenia and DUT mutations, and the fact that platelets are derived from megakaryocyte fragmentation, we further explored the relationship between DUT and megakaryocytes. Immunofluorescence analysis revealed a high degree of the co-localization of DUT with CD41+ cells (a specific marker for megakaryocyte differentiation and maturation) in the spleen of wild-type (WT) rabbits, indicating that DUT is highly expressed in megakaryocytes (Figure 2D). Given that CD41 expression increases with DNA ploidy during megakaryocyte differentiation, we used PMA-induced Meg-01 cells, a well-established model for studying megakaryocyte differentiation and polyploidization. Following PMA stimulation, DUT protein levels significantly increased by day 3 (Figure 2E,F), suggesting that DUT may be involved in the PMA-induced maturation and differentiation of megakaryocytes.

### 2.3. DUT Mutations Disruption of Platelet Function in Rabbits

**Figure 3:**
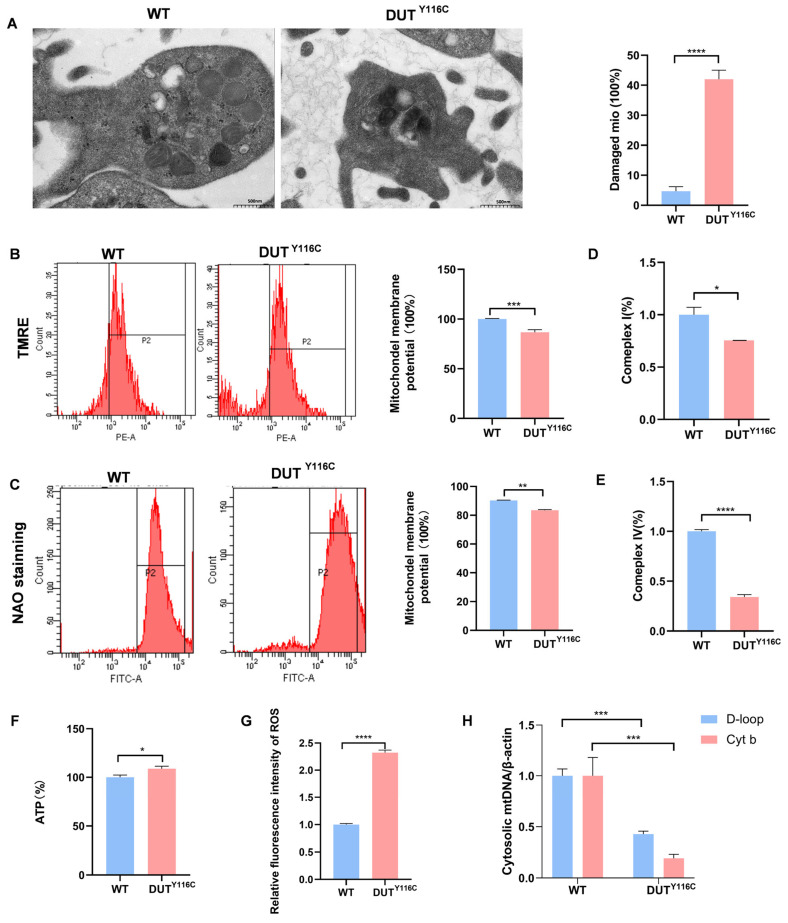
Mitochondrial dysfunction of platelets in DUT (p.Y116C) rabbits. Platelets were isolated from the blood of DUT (p.Y116C) rabbits and WT rabbits. (**A**) Platelets were detected by electron microscopy. Platelets were examined by electron microscopy. Each group consisted of 100 platelets, with a total of three groups (*n* ≥ 3; values are the means ± SEM; **** *p* < 0.0001). (**B**) Mitochondrial membrane potential was detected by flow cytometry using TMRE staining. Statistical analysis of the data is shown in panel (*n* ≥ 3; values are the means ± SEM; *** *p* < 0.001). (**C**) Platelets were treated with the selective mitochondrial dye nonyl acridine orange (NAO) and analyzed by flow cytometry to determine the mitochondrial mass. Statistical analysis of the data is shown in panel (*n* ≥ 3; values are the means ± SEM; ** *p* < 0.01). (**D**,**E**) Enzyme activity assay of mitochondrial complex I and IV (*n* ≥ 3; values are the means ± SEM; * *p* < 0.05, **** *p* < 0.0001). (**F**) Statistical analysis of the ATP production capacity of platelets from DUT (p.Y116C) rabbits and WT controls (*n* ≥ 3; * *p* < 0.05). (**G**) Statistical analysis of the ROS production capacity of platelets from DUT (p.Y116C) rabbits and WT controls (*n* ≥ 3; **** *p* < 0.0001). (**H**) Single-cell mtDNA quantification from platelets was isolated from the blood of DUT (p.Y116C) rabbits and WT rabbits (*n* ≥ 3; values are the means ± SEM; *** *p* < 0.001).

To further characterize the impact of DUT (p.Y116C) on platelet morphology, ultrastructural analysis by electron microscopy revealed significant alterations in the subcellular architecture of mutant platelets. Notably, mutant platelets exhibited impaired mitochondrial membrane integrity, deepening of the mitochondrial matrix, and disorganized ridge morphology (Figure 3A). Flow cytometry using TMRE, a selective marker for healthy mitochondria, showed reduced mitochondrial membrane potential in mutant platelets (Figure 3B). Nonyl acridine orange staining also revealed a decrease in mitochondrial mass in DUT (p.Y116C) platelets (Figure 3C). Functional assessments indicated impaired mitochondrial respiratory capacity, with reduced activity of mitochondrial complexes I and IV in DUT (p.Y116C) platelets compared to WT controls (Figure 3D,E). The analysis further confirmed elevated mitochondrial ROS levels and a slight increase in ATP production in mutant platelets (Figure 3F,G). In addition, quantification of mtDNA copy number showed a significant reduction in platelet mitochondrial mtDNA in mutant rabbits compared to WT (Figure 3H). Collectively, these findings demonstrate that the DUT (p.Y116C) mutation disrupts mitochondrial quality and functional integrity in platelets.

### 2.4. DUT Mutations Lead to Increased Mitophagy in Rabbits

**Figure 4:**
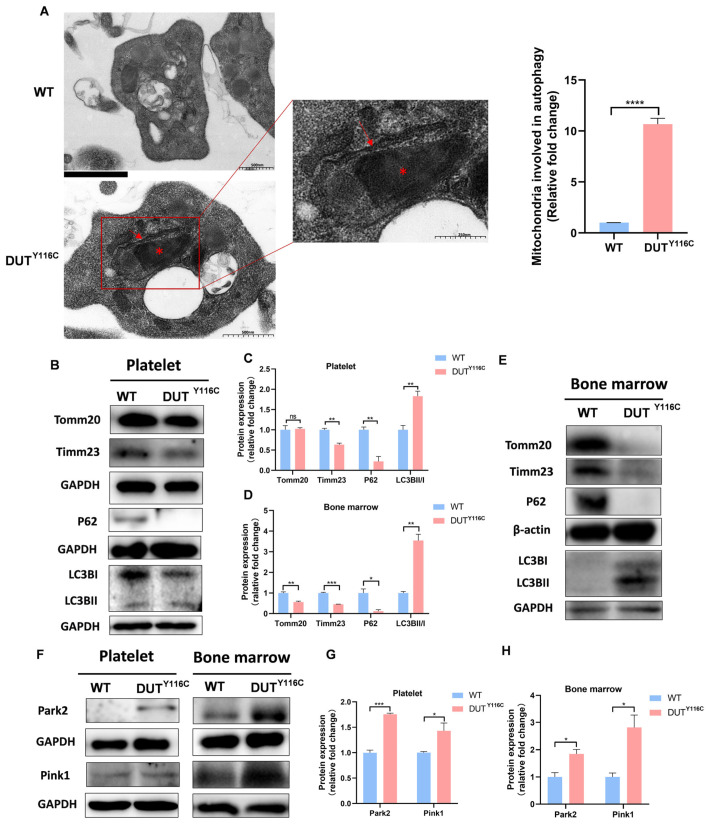
Increased mitophagy of platelets in DUT (p.Y116C) rabbits. (**A**) Platelets were detected by electron microscopy. Mitophagy phenomenon of mitochondria enclosed in autophagosomes was quantitated, and the data demonstrated that 7 of 500 platelets were observed within the autophagosomes in DUT (p. Y116C), and 1 of 800 platelets were observed within the autophagosomes in WT. Red asterisk, mitochondrion; red arrow, double-membrane autophagic structures of mitophagosomes (values are the means ± SEM, **** *p* < 0.001). (**B**,**C**) Tomm20, Timm23, P62, and LC3B levels were tested in the platelets of DUT (p.Y116C) rabbits and control rabbits by Western blot analysis. The grayscale values of the bands in platelets were measured with ImageJ software, and the statistical data are illustrated (*n* ≥ 3; values are the means ± SEM, an ANOVA was used, ns, not significant; ** *p* < 0.01). (**D**,**E**) Tomm20, Timm23, P62, and LC3B levels were tested in the bone marrow of DUT (Y116C) rabbits and control rabbits by Western blot analysis. The grayscale values of the bands in the bone marrow were measured with ImageJ software, and the statistical data are illustrated (*n* ≥ 3; values are the means ± SEM, an ANOVA was used, ns, not significant; * *p* < 0.05; ** *p* < 0.01; *** *p* < 0.001). (**F**–**H**) Park2 and Pink1 levels were tested in the platelets and bone marrow of DUT (Y116C) rabbits and control rabbits by Western blot analysis. The grayscale values of the bands in bone marrow were measured with ImageJ software and the statistical data are illustrated (*n* ≥ 3; values are the means ± SEM, an ANOVA was used, * *p* < 0.05; *** *p* < 0.001).

Mitophagy is a selective autophagic process that eliminates damaged mitochondria to maintain cellular homeostasis. Impaired mitophagy can lead to mitochondrial dysfunction. To assess whether the DUT (p.Y116C) mutation affects platelet mitophagy, we performed ultrastructural analysis using electron microscopy. Platelets from DUT (p.Y116C) rabbits exhibited characteristic mitophagy structures, including mitochondria enclosed within double-membrane mitophagosomes (Figure 4A). Correspondingly, the levels of autophagy-related proteins were significantly altered: the inner membrane protein Timm23 and autophagy adaptor protein P62 (SQSTM1) were decreased, while LC3BII/I levels were increased in DUT (p.Y116C) platelets. However, no significant changes were observed in the outer membrane protein Tomm20 (Figure 4B,C). Moreover, the DUT (p.Y116C) mutation reduced mitochondrial protein levels, including Timm23, Tomm20, and P62 in both spleen and bone marrow (Figure 4D,E and Figure S2). Western blot analysis revealed elevated levels of Park2 and Pink1 (Figure 4F–H), suggesting mitophagy activation via the Pink1-Park2 pathway. These findings collectively suggest that the DUT (p.Y116C) mutation significantly exacerbates mitophagy, highlighting its critical role in regulating platelet mitophagy.

## 3. Discussion

In 2017, Dos Santos et al. described a new autosomal recessive syndrome characterized by early-onset diabetes and bone marrow failure, including macrocytosis, anemia, and thrombocytopenia. This is the first report of a DUT mutation in humans [16]. In this study, we first present a new DUT (p.Y116C) thrombocytopenia rabbit model that mimics thrombocytopenia symptoms in human cases, providing a unique platform for elucidating the relevant pathogenesis mechanism.

Deoxyuridine 5′-triphosphate nucleotidohydrolase, dUTPase (DUT) plays a critical role in DNA replication and repair by ensuring the fidelity of DNA synthesis and maintaining genome stability. Recent studies have proposed that the redox status of cysteine residues functions as a redox switch, modulating both the oligomeric state and enzymatic activity of human dUTPase. However, contrasting findings indicate that the DUT (p.Y142C) mutation does not impair substrate binding or catalytic efficiency at room temperature, but significantly reduces the thermal stability of the enzyme [18,24]. In our study, it was found that the DUT (p.Y116C) mutation in rabbits resulted in a decreased protein expression of DUT in the spleen and platelets.

Thrombocytopenia is a common clinical condition. The main pathological causes of thrombocytopenia include a reduction in platelet production, platelet destruction, and excessive consumption [25]. However, the association of DUT with platelets and megakaryocytes is still unknown. In our study, we found that DUT was highly expressed in megakaryocytes and may be associated with megakaryocyte differentiation. In addition, thrombocytopenia and megakaryocyte reduction were found in DUT (p.Y116C)-mutant rabbits. These results suggest that DUT (p.Y116C) is associated with decreased platelet counts, but the underlying mechanism needs to be elucidated in future studies.

**Figure 5:**
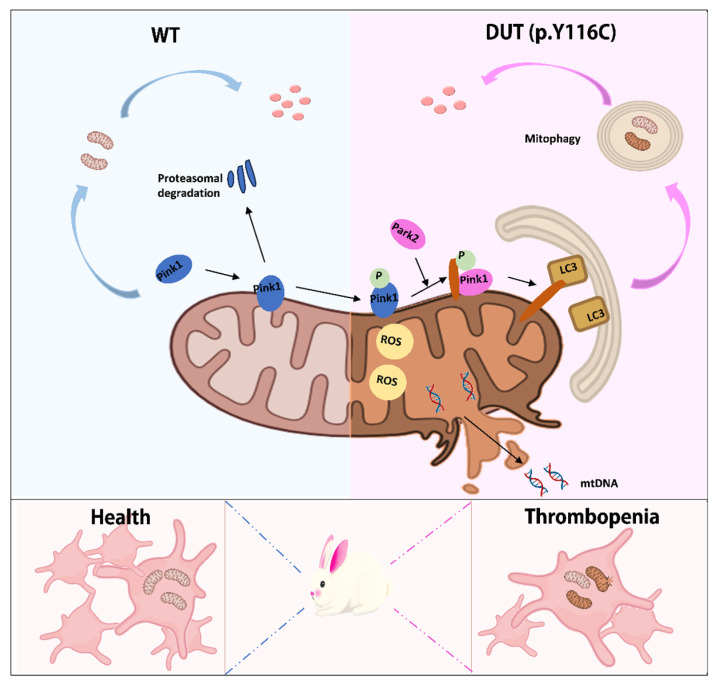
Platelets from DUT (p.Y116C)-mutant rabbits exhibited significant mitochondrial abnormalities associated with thrombocytopenia pathogenesis. The mitochondrial integrity of mutant rabbit platelets is impaired and the outer membrane structure is discontinuous. At the same time, these platelets exhibit abnormal alterations in mitochondrial function, including decreased mitochondrial DNA (mtDNA) and an abnormal accumulation of mitochondrial reactive oxygen species (ROS) levels. At the same time, platelet mitophagy increases.

Mitophagy is essential for preserving mitochondrial quality, energy metabolism, and organ function in cells rich in mitochondria, such as neurons, cardiomyocytes, and skeletal muscle cells [5,9]. However, the role of mitophagy in platelets, which possess a limited number of mitochondria, remains incompletely understood. In this study, rabbit DUT (p.Y116C) mutations resulted in impaired mitochondrial accumulation, excessive mitochondrial ROS production, and impaired platelet respiration, resulting in a decrease in mitochondrial membrane potential. DUT/Park2 may play an important role in mitophagy and apoptosis. Therefore, our findings suggest that DUT (p.Y116C) has an effect on controlling basal mitophagy, mitochondrial mass, and mitochondrial function in platelets (Figure 5).

In conclusion, the DUT (p.Y116C)-mutant rabbit model serves as a reliable and novel model for studying thrombocytopenia associated with DUT dysfunction. The pathogenesis of thrombocytopenia in this model is closely linked to enhanced platelet mitophagy. Through this model, we reveal that reduced DUT expression leads to the upregulation of Park2 and LC3B, driving increased mitophagy and subsequent mitochondrial dysfunction. Additionally, while preliminary evidence suggests that this mutation may also impact megakaryocytes, the specific mechanisms by which DUT (p.Y116C) influences megakaryocyte biology remain unclear and warrant further investigation in future studies.

## 4. Materials and Methods

### 4.1. Animals and Ethics Statement

The New Zealand white rabbits utilized in this study were procured from the Laboratory Animal Center of Jilin University, located in Changchun, China. All experimental procedures involving animals were conducted in strict accordance with the guidelines and standards approved by the Animal Welfare and Research Ethics Committee of Jilin University, ensuring compliance with ethical principles and animal welfare regulations (IACUC number:SY202310012).

### 4.2. Experimental Environment

The laboratory was equipped with a temperature-controlled HVAC system. During the 2–5 h experimental delay period, all procedures were conducted at room temperature (20–25 °C) or under temperature-controlled conditions, as required by specific experimental protocols.

### 4.3. Vector Construction and In Vitro Transcription

Vector construction and in vitro transcription were performed following established protocols [26]. The pCMV-T7-ABEmax (7.10)-SpRY-P2A-EGFP plasmids were acquired from Addgene (Watertown, MA, USA). In brief, for sgRNA production, two complementary DNA oligonucleotides were annealed at 95 °C for 5 min to form double-stranded DNA, which was subsequently cloned into a BbsI-digested pUC57-simple vector expressing Cas9, obtained from Addgene (Watertown, MA, USA).

### 4.4. Embryo Collection, Microinjection, and Transfer

The microinjection procedure for pronuclear-stage embryos followed our previously published protocols [26]. Briefly, a mixture of Cas9 mRNA (70 ng/μL) and sgRNA (35 ng/μL) was co-injected into the cytoplasm of pronuclear-stage zygotes, which were then transferred into the oviducts of surrogate rabbits.

### 4.5. Complete Blood Count

Blood samples were collected from the marginal ear artery of rabbits and immediately mixed with ACD anticoagulant (sodium citrate–dextrose solution). Platelet and other blood cell counts were performed using an automated hematology analyzer.

### 4.6. Platelet Preparation

Washed platelets were prepared as described previously [27]. Briefly, whole blood was collected from the auricular marginal artery of rabbits using an acid–citrate–dextrose (ACD) anticoagulant solution (2% dextrose, 1.5% citric acid, and 2.5% trisodium citrate) supplemented with 1 U/mL apyrase at a one-seventh volume ratio. Platelets were isolated following the manufacturer’s instructions (Beibo, BB-31410, China).

### 4.7. Western Blots

Total protein was extracted from tissues and cells using RIPA lysis buffer supplemented with PMSF (Beyotime, ST506, Shanghai, China) and phosphatase inhibitor (Meilunbio, MB12707-1, Dalian, China). Protein concentrations were determined using an enhanced BCA protein assay kit (Beyotime, P0010, Shanghai, China). Proteins were separated on 10% or 12% SDS-PAGE gels and transferred to 0.22 µm PVDF membranes (Boster, AR0137-04, Pleasanton, CA, USA). Membranes were blocked with 5% non-fat milk (Boster, AR0104, USA) and probed with specific antibodies. The antibodies used are listed in Table S1B.

### 4.8. Immunohistochemical Staining of Rabbits Femur and Spleen

The rabbits were anesthetized and sacrificed, and their femurs were fixed in 4% paraformaldehyde for 48 h. The femur tissues were then rinsed three times with PBS and distilled water, 20 min each. After decalcification, the femur and spleen tissues were processed using a tissue dehydration and embedding machine for dehydration, transparency, and paraffin embedding, followed by staining.

### 4.9. Platelet Annexin V Staining

The washed platelets were resuspended in annexin V binding buffer and incubated with annexin V-FITC (Meilunbio, MA0220-1, China) for 30 min. Samples were analyzed using a flow cytometer (BD, Biosciences, Torrance, CA, USA), and data were processed with FlowJo™ v10.10 software (FlowJo, Ashland, OR, USA).

### 4.10. Detection of TPO in Serum

Following blood collection from the auricular artery of rabbits, the samples were centrifuged at 3000 rpm for 10 min at room temperature. The supernatant was carefully aspirated and stored at −80 °C to prevent protein degradation in the serum due to repeated freeze–thaw cycles. Serum TPO concentrations were quantified using a TPO ELISA Assay Kit (Sinobestbio, YX-201615R, Shanghai, China).

### 4.11. Mitophagy Analysis by Electron Microscopy

Morphological mitophagy in platelets was assessed using electron microscopy. Platelets isolated from 3-month-old DUT (p.Y116C) and control rabbits were fixed with 2.5% glutaraldehyde for 120 min at room temperature. Mitophagy analysis was conducted as previously described, using a 120-kV JEOL electron microscope operating at 80 kV (JEOL Ltd., Akishima, Japan).

### 4.12. Mitochondrial Inner Transmembrane Potential and Mitochondrial Mass Assay

The mitochondrial inner transmembrane potential in pretreated platelets was assessed by flow cytometry using tetramethylrhodamine ethyl ester perchlorate (TMRE) (Meilunbio, C2001s, China). Similarly, mitochondrial mass was evaluated using the mitochondrial dye nonyl acridine orange (NAO) (Biodee, BN14002, Beijing, China) via flow cytometry.

### 4.13. ATP and ROS Assay

Intracellular ATP generation was detected using an ATP detection kit (Beyotime, S0026, China). Intracellular ROS generation was detected using an ROS detection kit (Abbkine, KTB1911, Wuhan, China).

### 4.14. mtDNA Analysis

Mitochondrial DNA (mtDNA) was quantified with quantitative PCR. For the rabbit mtDNA, the mitochondrialcytochrome b (Cytb) gene was amplified by PCR with the following primers: forward 5-TCTACATACACGTAGGCCGCGGAA-3 and reverse 5-GAGGAGAAGAATGGCTA-CAAGGAAA-3. The D-loop gene was amplified with the following primers: forward 5-TGAATCGGAGGCCAACCAGTA-3 and reverse 5-CATCGAGATGTCTTATTTAAG-3 [28]. The ACTB gene was used as housekeeping gene with the following primers: forward 5-CTGGAACGGTGAAGGTGACA-3 and reverse 5- CGGCCACATTGCAGAACTTT-3 [29].

## Data Availability

Data will be made available on request.

## Supplementary Materials

The following supporting information can be downloaded at: https://www.mdpi.com/article/10.3390/ijms26094169/s1.
