# Supplementary material for: DUT (p.Y116C)-Mutation-Induced Thrombocytopenia in Rabbits
Source: Int J Mol Sci. 2025 Apr 28;26(9):4169. doi: 10.3390/ijms26094169 (PMC12072063; doi:10.3390/ijms26094169)
Supplement: Supplementary file 1 [file ijms-26-04169-s001.zip › ijms-3514287-supplementary.pdf]

Figure S1

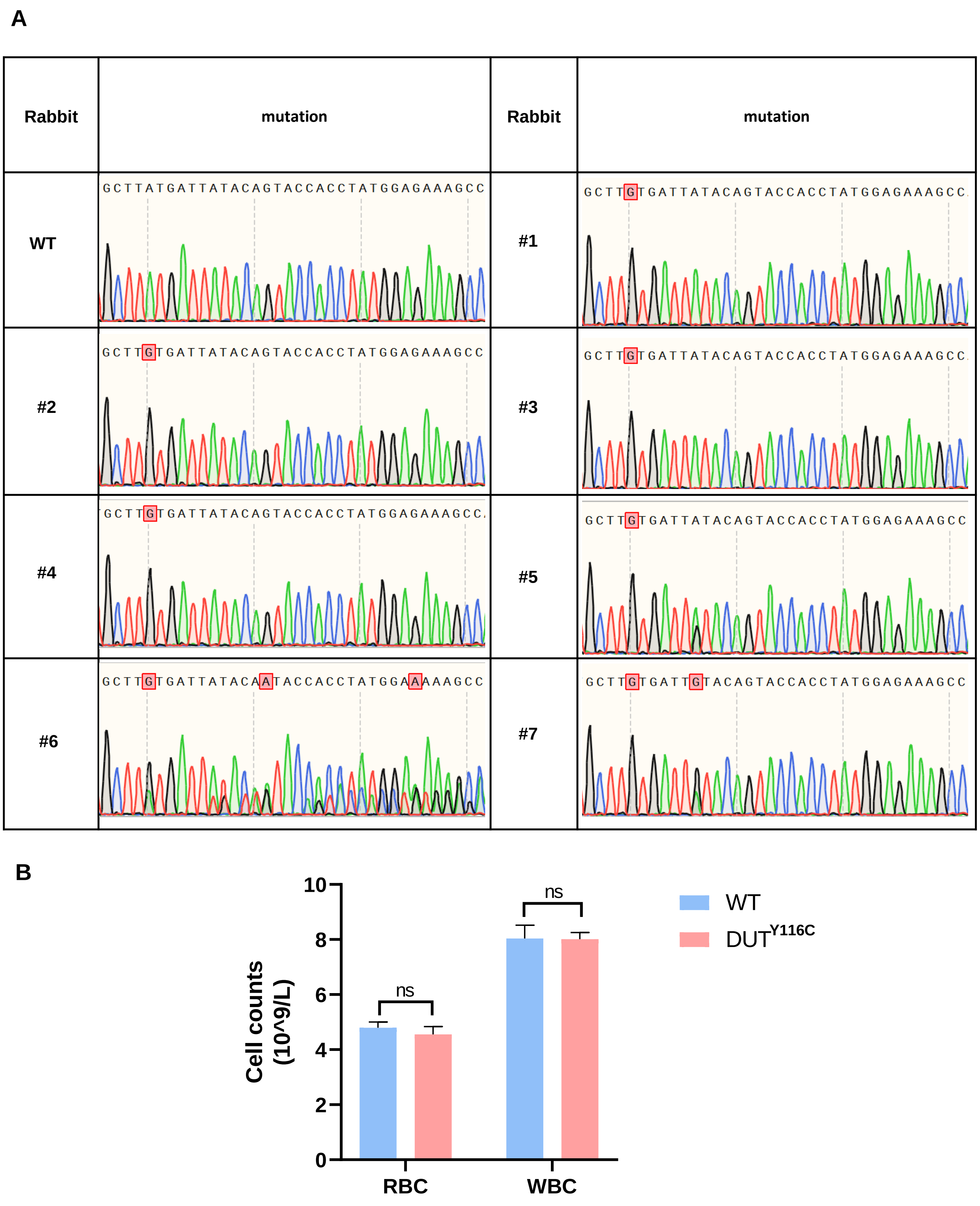

Figure S1. (**A**)Sanger sequencing results of DUT gene-edited rabbits. (**B**) RBC and WBC in WT and *DUT* (p.Y116C) rabbit (n⩾3; Values are means ± SEM, ns, not significant).

Figure S2

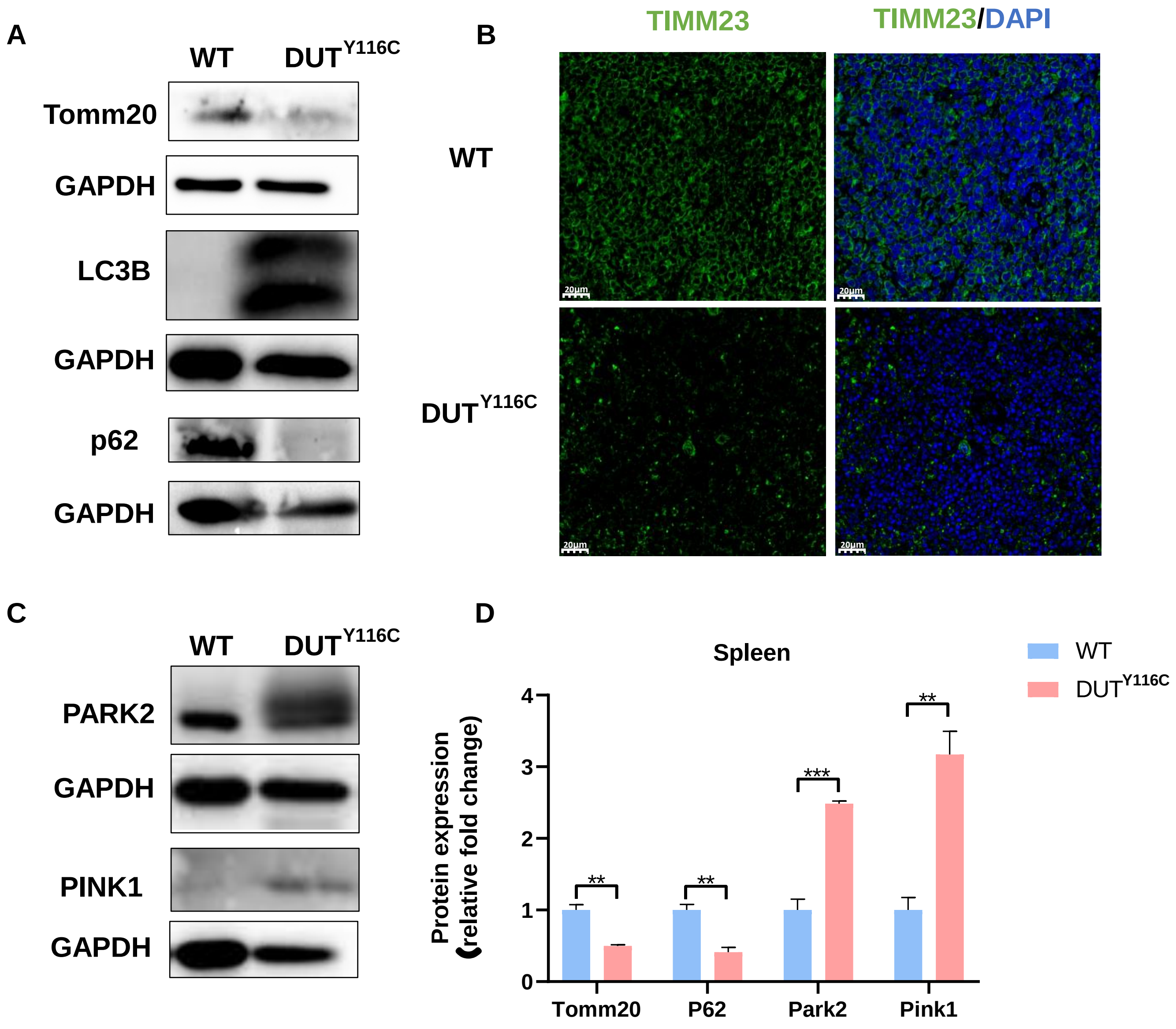

Figure S2. (**A**) Tomm20 and LC3B and P62 levels were tested in spleen of DUT (Y116C) rabbits and control rabbits by western blot analysis. (**B**)Immunolabeling of Timm23(green) and DAPI (blue) in spleen of DUT (Y116C) rabbits and WT. （**C**） Park2 andPink1 levels were tested in spleen of DUT (Y116C) rabbits and control rabbits by western blot analysis. (**D**) The grayscale values of the bands in spleen were measured with ImageJ software and the statistical data are illustrated. (n⩾3; Values are means ± SEM , ANOVA was used; **P < .01； ***P < .001).

Figure S3

A.The Oligonucleotide used for sgRNA and PCR

Name	Primers	Sequence (5’-3’)
Rabbit DUT sgRNA	DUT-O-sgRNA	GCTTGTGATTATACAGTACCACCT
Rabbit DUT primers	DUT-O-F	GCATTTCTGTGCTTGGTCAC
	DUT-O-R	CATCCAGAAGGAAGAGCTATCTG

B.The antibodies used in this study

Antibody	Company	Cat No.	Source
P62	Zen-Bioscience	382862	Rabbit
DUT	Proteintech	13740-1-AP	Rabbit
PINK1	Proteintech	23274-1-AP	Rabbit
CD41	Abconal	A11490	Rabbit
TOMM20	Abconal	A19403	Rabbit
TIMM23	Abconal	A8688	Rabbit
PARK2	Proteintech	14060-1-AP	Rabbit
LC3B	Starter	S0B0404	Rabbit
GAPDH	Proteintech	60004-1-Ig	Mouse
GAPDH	Proteintech	10494-1-AP	Rabbit
